# Quantitation of glutathione *S*-transferases in rice (*Oryza sativa* L.) roots exposed to cadmium by liquid chromatography–tandem mass spectrometry using isotope-labeled wing peptides as an internal standard

**DOI:** 10.1186/s13007-017-0214-2

**Published:** 2017-08-04

**Authors:** Zhenzhen Cao, Renxiang Mou, Zhaoyun Cao, Xiaoyan Lin, Youning Ma, Zhiwei Zhu, Mingxue Chen

**Affiliations:** Rice Product Quality Supervision and Inspection Center, China National Rice Research Institute, Hangzhou, 310006 China

**Keywords:** Glutathione *S*-transferases, Cadmium, Liquid chromatography–tandem mass spectrometry, Signature peptide, Isotope-labeled internal standard

## Abstract

**Background:**

Plant glutathione *S*-transferases (GSTs, EC 2.5.1.18) are multifunctional enzymes involved in heavy metal cellular detoxification by conjugating the tripeptide (g-Glu-Cys-Gly) glutathione to heavy metals. Previous studies demonstrated that individual rice GSTs were differentially induced by heavy metal exposure at the mRNA transcript level. However, little information is available concerning changes in protein concentration of rice GSTs under heavy metal stress. Because the correlation between changes in protein concentration and gene expression under abiotic stress is poor, direct determination of rice GSTs protein concentrations during cadmium (Cd) exposure is a more effective and reliable approach to explore possible mechanisms of rice Cd translocation and accumulation.

**Results:**

This study established an optimized and advanced liquid chromatography–tandem mass spectrometry (LC–MS/MS)-based targeted proteomics assay for quantification of OsGSTF14 and OsGSTU6 proteins in Cd-stressed rice roots. The tryptic signature peptides were chosen as surrogate analytes and winged peptides containing the isotope-labeled signature peptides were used as the internal standards. The signature peptides exhibited good linearity in the range of 0.6–60 and 0.3–30 nM, respectively. The limit of detection and limit of quantification were 4.5 and 14.5 µg/g for OsGSTF14, respectively, and 2.1 and 7.0 µg/g for OsGSTU6. The spiking recoveries rates at low, medium and high levels were in the range of 72.5–93.4%, with intra- and inter-day precisions of 5.5–9.1 and 4.2–10.2%, respectively.

**Conclusions:**

The assay successfully quantified the temporal and dose responses of OsGSTF14 and OsGSTU6 proteins in Cd-stressed rice roots, with good accuracy, precision and high-throughput. This assay will have significant application in developing quantification methods of other proteins in Cd-stressed rice, which may provide more insight into the mechanisms of Cd translocation and accumulation in rice.

**Electronic supplementary material:**

The online version of this article (doi:10.1186/s13007-017-0214-2) contains supplementary material, which is available to authorized users.

## Background

Cadmium (Cd) is an extremely toxic heavy metal and recognized as one of the important environmental pollutants in agricultural soil [1, 2]. Cd accumulation in agricultural soil can potentially cause harmful effects to humans through its uptake and accumulation by food crops from Cd-contaminated soil [3, 4]. Rice (*Oryza sativa* L.), as an important staple food for Asian population, has been identified as the major source of Cd intake from food consumption. Approximately 40–50% of the daily intake of Cd for Japanese and Chinese populations was from rice [5, 6]. Thus, it is urgent to elucidate the mechanisms of rice Cd translocation and accumulation to develop strategies to prevent its accumulation in rice grains, so as to reduce potential health risks to humans from rice consumption.

Glutathione *S*-transferases (GSTs, EC 2.5.1.18) are multifunctional enzymes involved in plant cellular detoxification by conjugating the tripeptide (g-Glu-Cys-Gly) glutathione (GSH) to a wide variety of substrates such as endobiotic and xenobiotic compounds [7, 8]. As a detoxification mechanism, such S-glutathionylated conjugate metabolites are compartmentalized in plant vacuoles, thereby reducing translocation and accumulation of toxic compounds in plants. Based on protein homology and gene organization, plant GSTs can be categorized into five subclasses: phi (GSTF), zeta (GSTZ), tau (GSTU), theta (GSTT) and lambda (GSTL) [9–11]. Among these, tau and phi subclasses are plant-specific, and are most widely studied in plants. Plant GSTs were first identified and have been intensively studied with regard to herbicide detoxification [12–14]. Recently, it was demonstrated that individual plant GSTs were differentially induced by heavy metal exposure. For example, Chakrabarty et al. [15] and Dubey et al. [16] reported that *OsGSTU6* was specifically up-regulated in arsenate (AsV) and chromium (Cr)-stressed rice roots and suggested the role of *OsGSTU6* in the AsV and Cr detoxification mechanisms. Similarly, Jain et al. [17] showed that *OsGSTF5* was AsV stress-induced and differentially expressed in AsV-sensitive and AsV-resistant rice roots during AsV stress, indicating that *OsGSTF5* possibly regulates rice tolerance to AsV stress. These studies significantly improved our understanding of diversity and functions of individual rice GSTs in response to heavy metal stress at the mRNA transcript level. However, little information is available for changes in protein concentration of rice GSTs under heavy metal stress. The correlation between changes in protein concentration and gene expression under abiotic stress is poor due to post-translational modifications [18, 19], and gene expression levels from mRNA transcript analysis are insufficient to predict protein concentration changes in response to abiotic stress. Thus, direct determination of rice GSTs protein concentrations during Cd exposure is a more effective and reliable approach to explore the possible mechanisms of rice Cd translocation and accumulation.

Traditional analytical methods for determination of plant proteins include 2D-gel electrophoresis and immunoassays, such as western-blot analysis and enzyme-linked immunosorbent assay (ELISA). 2D-gel electrophoresis is a powerful tool for separation and visualization of the interest proteins, but the technique always misses some important protein information, such as low abundance proteins and hydrophobic proteins; additionally, it is time-consuming, laborious and lacks sensitivity [20, 21]. Western-blot and ELISA methods are widely accepted as highly sensitive and selective tools for target protein quantification. However, their effectiveness is often strongly correlated with the quality of antigen and antibodies, and the generation of high-specificity antibodies for plant proteins is both challenging and time-consuming. In addition, nonspecific binding of the antibody or antigen to the plate will cause a falsely high-positive result [22, 23]. These disadvantages limit their large-scale application in protein quantification. In recent years, protein quantitative approach based on liquid chromatography–tandem mass spectrometry (LC–MS/MS) has become increasingly popular in target-proteomics studies, due to good accuracy, precision and high-throughput for protein quantification in high-complexity samples [24–26]. Multiple reaction monitoring mass spectrometry incorporated with stable isotope-labeled synthetic internal standard peptide is one of the LC–MS/MS-based protein quantitative approaches. In this approach, a tryptic signature peptide for protein is synthesized in an isotope-labeled form and added to the biological sample at a known concentration, and protein is quantified by comparing ion signals from native and isotope-labeled peptides after protein digestion. To date, this approach has been widely applied in disease biomarker quantification studies [27–29]. For example, Yang et al. [27] employed stable isotope-labeled peptide for quantification of p-glycoprotein (P-gp) levels in the breast cancer drug-sensitive and resistant cells, and Fallon et al. [29] quantified four efflux drug transporters in liver and kidney across species using stable isotope-labeled peptide. As reported, these assays have greater sensitivity and specificity than the immunoassays. However, it is noteworthy that the synthetic isotope-labeled internal standard peptides used in these studies are added after digestion and thus the variations that occur during sample extraction and protein digestion are neglected. Thus, selection and synthesis of isotope-labeled internal standard peptides have to been further optimized to avoid these variations. Additionally, quantification of target proteins in response to various abiotic stresses using LC–MS/MS-based protein quantitative assay has been rarely reported in rice plants, due to the fact that rice plants always contain large amounts of secondary compounds that interfere with protein extraction and digestion, which restricts development of targeted proteomics in plants.

In the present work, an optimized and advanced LC–MS/MS-based targeted proteomics assay for quantification of two GST members (OsGSTF14 and OsGSTU6) in rice roots was developed and validated. OsGSTF14 and OsGSTU6 were selected based on their significant responses to heavy metal stress according to previous studies [15–17]. A stable isotope-labeled synthetic winged peptide was used as an internal standard. Finally, this approach was applied in determination of the temporal and dose responses of OsGSTF14 and OsGSTU6 proteins in Cd-stressed rice roots. This assay will be useful for developing quantification methods of other proteins in Cd-stressed rice, which may provide more insight concerning the mechanisms of Cd translocation and accumulation in rice.

## Methods

### Chemicals and reagents

Urea, ammonium bicarbonate (NH_4_HCO_3_), dithiotheritol (DTT), iodoacetamide (IAA) and calcium chloride (CaCl_2_) were analytical grade and obtained from Sigma-Aldrich (St. Louis, MO, USA). Sequence grade modified trypsin was purchased from Promega (Madison, WI, USA). Formic acid (FA) and acetonitrile (ACN) of HPLC grade were obtained from Merck (Darmstadt, Germany). Deionized water (18.2 MΩ) was produced by a Milli-Q water purification system (Millipore Co., Bedford, MA, USA) and used throughout all analyses.

### OsGSTU6 and OsGSTF14 protein standard expression and purification

Rice full-length cDNAs encoding OsGSTF14 and OsGSTU6 (LOC_Os03g04220.1and LOC_Os10g38700) from the Rice Genome Annotation Project Database were synthesized and cloned into the pET-30a (+) expression vector. The constructed recombinant plasmids were transformed into *E. coli* BL21 (DE3) and grown in LB medium containing kanamycin at 37 °C until the cell density reached 0.6–0.8 at 600 nm. Protein expression was induced by addition of 1 mM isopropyl β-D-1-thiogalactopyranoside (IPTG). After 4 h of induction, the cells were collected by centrifugation at 10,000*g* for 10 min, resuspended in 50 mM Tris–HCl (pH 8.0) and 150 mM NaCl, and then sonicated. After centrifugation at 20,000*g* for 20 min, the supernatants containing OsGSTU6 and OsGSTF14 proteins were purified by one-step purification using Ni column and eluted in buffer containing 50 mM Tris–HCl (pH 8.0), 300 mM NaCl and 250 mM imidazole. Protein concentrations were determined using the BCA protein assay kit (Pierce, Rockford, IL, USA). Protein expression and purification were evaluated with SDS-PAGE and western blot (Additional file 1).

### Synthetic peptide standards

The signature peptide VFGSPTSAEVAR for OsGSTF14, TPLLAAWAER for OsGSTU6, stable isotope-labeled signature peptides VFGSPTSAEV*(Val-OH-13C5,15N)AR, TPLLAAWAER*(Arg-OH-13C6,15N4), and internal standard peptides APASVKVFGSPTSAEV*(Val-OH-13C5,15N)ARVLMCLF, LVDAGKTPLLAAWAER*(Arg-OH-13C6,15N4)FVEVEA were synthesized by ChinaPeptides Co., Ltd. (Shanghai, China). All peptide standards were synthesized with purity of more than 95%. A 1 mg/mL stock solution was prepared by accurately weighing the peptide and dissolving it in an ACN-ultrapure water mixture (20:80, v/v).

### Rice culture and total protein extraction

The hydroponic experiments were conducted during rice-growing seasons during 2015. The *indica* rice genotype (*cv*.9311) was used for this study, which was a well-known commercial indica rice cultivars and now available for its full genome sequencing and annotation by international rice genome project (http://rise2.genomics.org.cn/). Rice seeds were sterilized with 10% sodium hypochlorite (NaClO) (v/v) solution for 20 min, thoroughly washed with deionized water, soaked in deionized water for 2 days, and then germinated on moist filter paper at 37 °C for 1 day. The germinated seeds were grown in sterilized moist quartz sand under a controlled chamber at 28/22 °C (day/light temperatures, respectively), with a 16-h light/8-h dark photoperiod, light intensity of 225 ± 25 mol m^−2^ s^−1^ and 85% relative humidity. Ten-day-old rice seedlings with uniform size were selected and transplanted into a 5-L plastic container containing Yoshida’s culture solution [30]. After the third leaf was fully expanded, the rice seedlings were transferred to fresh nutrient solutions containing CdCl_2_ at different concentrations of 0, 0.5, 5 and 50 μM (three replicates per treatment). Rice roots were harvested after 5, 10 and 15 days of treatment, washed with deionized water, immediately frozen in liquid nitrogen and stored at −80 °C for protein and RNA extraction.

For total protein extraction, 1.0 g of frozen rice roots of different treatments was ground to fine powder under liquid nitrogen with a mortar and pestle. The powder was suspended in lysis buffer containing 8 M urea, 10 mM DTT, 1 mM phenylmethyl sulfonyl fluoride (PMSF), 2 mM EDTA and 20 mM Tris–HCl (pH 8.5). After centrifugation at 20,000*g* for 30 min, the protein concentrations of the supernatants were determined using the BCA protein assay kit (Pierce, Rockford, IL, USA).

### Preparation of tryptic hydrolysates

Prior to tryptic hydrolysis, 100 µL of protein lysis buffer containing approximately 150 µg of total protein was spiked with 10 µL of 4 µM internal standard peptide APASVKVFGSPTSAEV*ARVLMCLF and 10 µL of 2.5 µM internal standard peptide LVDAGKTPLLAAWAER*FVEVEA, respectively, and then mixed with 900 µL of 50 mM NH_4_HCO_3_. The mixtures were reduced with 10 mM DTT solution at 37 °C for 1 h and alkylated with 50 mM IAA in the dark for 45 min. Finally, 1 mM CaCl_2_ solution and sufficient sequencing grade trypsin solution (trypsin to protein ratio of 1:20, w/w) were added, and the mixtures were incubated at 37 °C for 16 h. The reaction was stopped by adding 1.0% FA.

The digested peptide mixtures were desalted on a MILI-SPE Extraction Disk Cartridge (C18-SD, Agilent Technologies, Palo Alto, CA, USA) according to Wisniewski et al. [31]. The eluate containing desalted peptides was diluted using ACN-ultrapure water (10:90, v/v) with 0.1% FA and centrifuged at 15,000*g* for 10 min at room temperature. The supernatant was analyzed by LC–MS/MS after passing through a 0.22-mm nylon filter.

### LC–MS/MS conditions

The LC was carried out using the Nexera LC-20ADXR HPLC System equipped with DGU-20A3R degassing unit, LC-20ADXR binary solvent delivery unit, SIL-20ACXR autosampler and CTO-20AC prominence column oven (Shimadzu, Columbia, MD, USA). The separation of peptides was performed with an Agilent Poroshell 120EC-C18 column (2.1 mm × 150 mm, 2.7 μm, Agilent, USA) at a flow rate of 0.2 mL/min. The injection volume was 5 µL and the column temperature was maintained at 40 °C. The mobile phase consisted of ultrapure water containing 0.1% FA (Solvent A) and acetonitrile containing 0.1% FA (Solvent B). The program used for elution was: 0–5 min, linear gradient 10–90% B; 5–9 min, isocratic 90% B; and 9.1 min, 10% B. The column was equilibrated for 9 min before the next injection.

The MS detection was performed using a QTRAP 5500 mass spectrometer equipped with a Turbo V electrospray source (AB Sciex, Foster City, CA, USA). The mass spectrometer was operated under the electrospray positive ion (ESI^+^) mode. Nitrogen was used as curtain and desolvation gas at the respective pressure of CUR: 35 psi, GS1: 40 psi and GS2: 40 psi. Source temperature was maintained at 450 °C, with the voltages for ISV of 5500 V and for EP of 10 V. Analytes were monitored in the multiple reaction monitoring (MRM) mode. All mass transitions, as well as their optimal CE,DP, CXP and EP during the MRM analysis are shown in Table 1. Data were processed using Analyst software (version 1.6.2; AB Sciex, Foster City, CA, USA).

**Table 1:** MS parameters on the precursor and product ion transitions for signature peptides, isotope-labeled signature peptides and internal standard peptides

Protein	Name	Sequence	Precursor ion (m/z)	Q1 mass (amu)	Q3 mass (amu)	DP (V)	CE (V)	CXP (V)	EP (V)
OsGSTF14	Signature peptide 1	VFGSPTSAEVAR	[M + 2H]^2+^	610.9	830.4	84.20	28.98	9.69	10
	Isotope-labeled signature peptide 1	VFGSPTSAEV*AR	[M + 2H]^2+^	613.9	836.4	95.97	29.30	9.46	10
	Internal standard peptide 1	APASVKVFGSPTSAEV*ARVLMCLF	[M + 3H]^3+^	829.7	987.6	106.17	28.84	7.46	10
OsGSTU6	Signature peptide 2	TPLLAAWAER	[M + 2H]^2+^	564.3	703.3	83.98	29.74	6.85	10
	Isotope-labeled signature peptide 2	TPLLAAWAER*	[M + 2H]^2+^	569.3	713.3	104.65	30.15	6.85	10
	Internal standard peptide 2	LVDAGKTPLLAAWAER*FVEVEA	[M + 3H]^3+^	799.3	1039.8	89.30	27.19	6.90	10

*DP* declustering potential, *CE* collision energy, *CXP* collision cell exit potential, *EP* entrance potential

### Method validation

The established method was validated by evaluation of matrix effects, linearity, sensitivity, recovery and precision (intra- and inter-day). Matrix effects were assessed by comparing responses of the signature peptides dissolved in neat solution to responses of the signature peptides spiked into sample extract. Linearity of two signature peptides was evaluated with the six different concentrations in the range of 0.6–60 and 0.3–30 nM (each containing 6 and 3 nM stable isotope-labeled signature peptide) by the internal standard method, respectively. Sensitivity was determined by evaluation of the limit of detection (LOD, signal-to-noise ratio ≥3) and the limit of quantitation (LOQ, signal-to-noise ratio ≥10). Recovery studies were performed by employing the standard addition method. Rice protein lysis buffer with internal standard was spiked with OsGSTU6 and OsGSTF14 standard protein at low (equal to the original level), medium and high levels, respectively. The recovery rates were calculated as the result of the measured value minus the original level divided by the spiked value. Intra- and inter-day precision (RSD) of the method were determined by recovery experiments at low, medium and high levels (n = 6) on 6 consecutive days.

### Statistics

Data were expressed as mean ± standard error of three biological and two technical replicates for each sample. Statistical analyses were performed using the statistical software OriginPro 8 SR0 (OriginLab Corporation, Northhampton, MA, USA). Statistical significance was assessed using Duncan’s multiple comparison at *P* < 0.05 level.

## Results and discussion

### Selection and synthesis of signature peptide standard and isotope-labeled signature peptide for OsGSTF14 and OsGSTU6

The most critical step of MS-based protein identification and quantitation using tryptic peptides is the selection of suitable tryptic signature peptides. The candidate signature peptides should be specific to the target protein and avoid containing amino acids susceptible to chemical modifications, such as cysteine and methionine. Additionally, the selected signature peptides should have high MS signal intensity and can be reproducibly observed in digested samples [24–26]. In the present work, full scan LC–MS/MS analyses for the tryptic peptides from standard proteins (OsGSTF14 and OsGSTU6) and rice root samples using nanoLC-LTQ-Orbitrap were performed. The signature peptides for OsGSTF14 and OsGSTU6 proteins were identified by comparing the endogenous and theoretical peptides from tryptic OsGSTF14 and OsGSTU6 proteins. The theoretical tryptic peptides of OsGSTF14 and OsGSTU6 proteins were obtained by computational prediction using online PeptideMass tools (http://web.expasy.org/peptide_mass/). According to the nanoLC-LTQ-Orbitrap analysis and sequence database search, five peptides were identified from standard proteins (OsGSTF14 and OsGSTU6) and rice root samples. The doubly charged ions of VFGSPTSAEVAR (residues 8–19) and TPLLAAWAER (residues 194–203) were selected and synthesized as the signature peptide of OsGSTF14 and OsGSTU6, respectively, due to their highest signal intensity and good reproducibility in rice root samples. The BLAST search results in NCBI (https://blast.ncbi.nlm.nih.gov/Blast.cgi) showed that the sequences of two selected signature peptides were unique to OsGSTF14 and OsGSTU6, respectively. Moreover, the signature peptides VFGSPTSAEVAR and TPLLAAWAER were also confirmed to be absent in the undigested rice sample matrices by mass spectrometry analysis, suggesting that they could be used to specifically quantify OsGSTF14 and OsGSTU6 proteins.

**Fig. 1:**
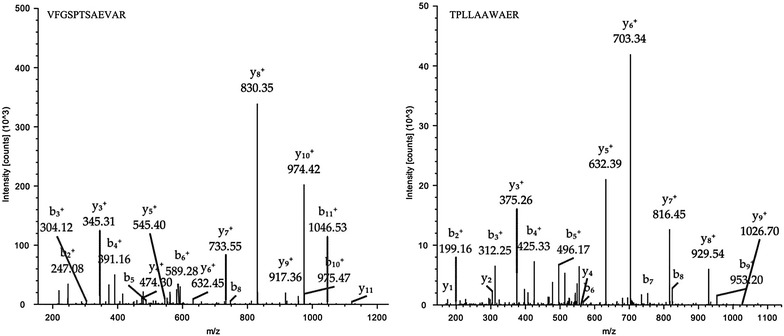
The product ion spectra of signature peptides VFGSPTSAEVAR and TPLLAAWAER

The product ion spectra of the signature peptides VFGSPTSAEVAR and TPLLAAWAER from LTQ-Orbitrap analysis are shown in Fig. 1. The highest abundance of y8 (m/z 830.4) and y6 fragment ions (m/z 703.3) were detected for signature peptides VFGSPTSAEVAR and TPLLAAWAER, respectively. Furthermore, the product ion spectra of synthetic signature peptides standard were further validated and optimized in QTRAP 5500 mass spectrometer (data not shown). The y8 ion of VFGSPTSAEVAR and y6 ion of TPLLAAWAER also showed relatively high abundance compared with the other fragment ions, which was consistent with LTQ-Orbitrap analysis results. Therefore, m/z 610.9 > 830.4 and 564.3 > 703.3 were selected as the quantitative analysis of ion pairs for the signature peptides VFGSPTSAEVAR and TPLLAAWAER, corresponding to y8 and y6 fragment ions, respectively.

**Fig. 2:**
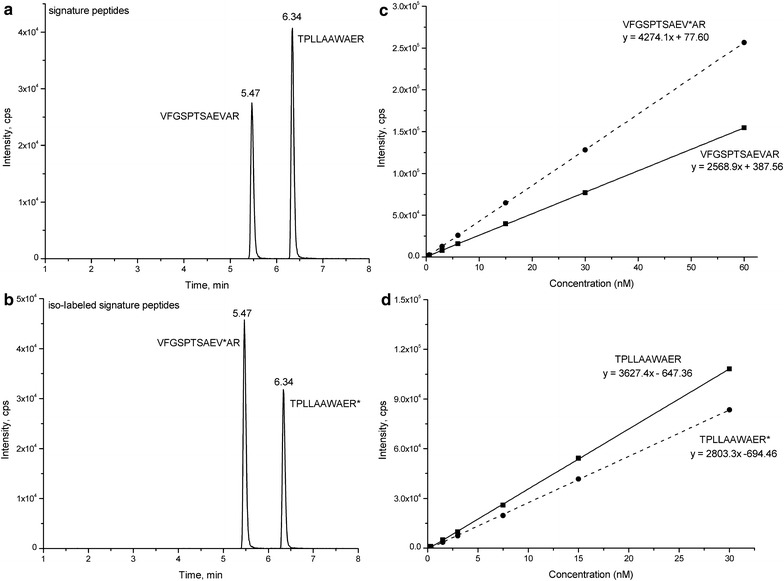
The LC–MS/MS chromatograms (**a**, **b**) and linear responses (**c**, **d**) of the signature peptides VFGSPTSAEVAR, TPLLAAWAER and their corresponding isotope-labeled signature peptides VFGSPTSAEV*AR, TPLLAAWAER*

Two stable isotope-labeled synthetic peptides, VFGSPTSAEV*AR and TPLLAAWAER*, were prepared. The stable isotope-labeled peptides showed added mass of 6 and 10 Da in y8 and y6 fragment ions compared with their non-labeled peptides, respectively, because of the isotope-labeled valine and lysine residues. The chromatograms and linear responses of the signature peptides and their corresponding isotope-labeled peptides during LC–MS/MS analysis are shown in Fig. 2. The retention time for the signature peptides and their corresponding isotope-labeled peptides were identical, with 5.47 ± 0.05 min (n = 20) for VFGSPTSAEVAR and VFGSPTSAEV*AR, and 6.34 ± 0.08 min (n = 20) for TPLLAAWAER and TPLLAAWAER* (Fig. 2a, b). Moreover, the signature peptides exhibited similar linearity responses to their corresponding isotope-labeled peptides, and linear response ratios of the signature peptides to the isotope-labeled peptides were in the range of 0.7–1.2 (Fig. 2c, d), suggesting that the isotope-labeled peptides have the similar chromatographic retention behavior and linear responses to their signature peptides, and could be used as internal standards for quantitative analysis of OsGSTF14 and OsGSTU6 proteins.

### Optimization and synthesis of internal standards

LC–MS/MS has developed into a powerful tool for protein quantification with high sensitivity and specificity. However, its accuracy is prone to be affected by ion suppression resulting from sample matrix or coeluting compounds [32]. To avoid the uncertainty induced by the variations in ion suppression, the most commonly used method is adding a known concentration of isotope-labeled internal standard peptides to the protein digests before LC–MS/MS analysis. The area ratio of the native peptide to the isotope-labeled peptide is used to estimate the target protein amount [25, 33]. This approach can effectively correct for ion suppression that occurs during LC–MS/MS analysis, and has high sensitivity and specificity in determining the target protein amount. However, it does not reflect the true expression levels of protein in the original sample because the internal standard is added after digestion and thus the variations that occur during sample preparation and protein digestion are neglected [25]. To alleviate this problem, in the present work, we employed a novel approach with a winged peptide as an internal standard to minimize the ionization efficiency and digestion variability. The winged peptide sequences were APASVKVFGSPTSAEV*ARVLMCLF and LVDAGKTPLLAAWAER*FVEVEA. They contained the isotope-labeled signature peptides and six amino acids natural flanking sequences around the cleavage sites of the signature peptides. The length of natural flanking sequences was selected according to Scott et al. [34], who found that synthetic signature peptides with six amino acids natural flanking sequences could significantly improve the signature peptide release from the native protein. The winged peptides were combined with the target protein at the beginning of the analytical process, thus could effectively correct for the variations that occur during sample preparation and protein digestion in the high-complexity rice samples.

### Tryptic digestion efficiency

**Fig. 3:**
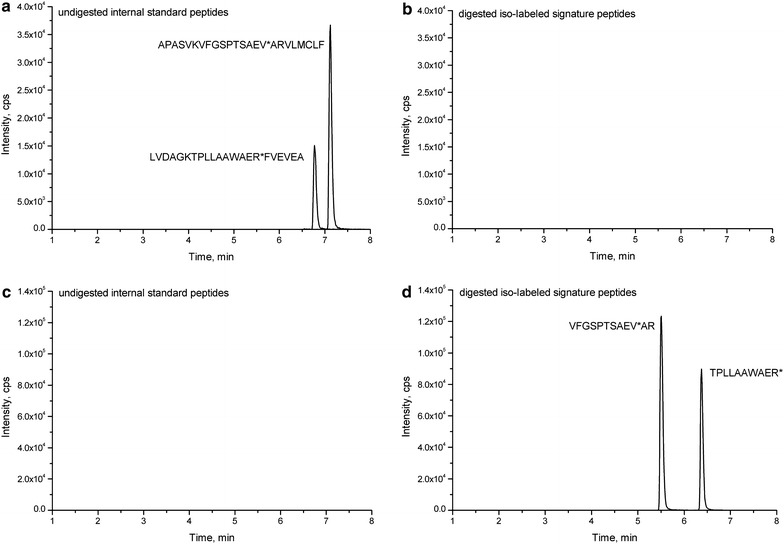
The LC–MS/MS chromatograms of internal standard peptides APASVKVFGSPTSAEV*ARVLMCLF, LVDAGKTPLLAAWAER*FVEVEA before (**a**, **b**) and after (**c**, **d**) tryptic digestion. The estimated amount of peptides on column is 0.5 µg

**Fig. 4:**
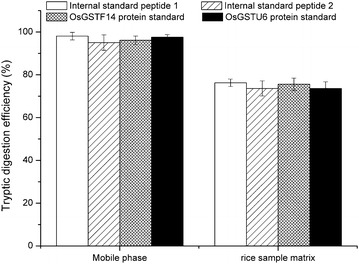
Tryptic digestion efficiency of standard proteins or internal standard peptides in different matrices

The standard proteins or internal standard peptides were employed to investigate the tryptic digestion efficiency of the target protein. The standard proteins or internal standard peptides were spiked into rice protein lysis buffer and digested using the same digestion protocol as described in the Methods section. Both the undigested internal standard peptides and the peptides after digestion were monitored using LC–MS/MS (Fig. 3). The analysis results showed that the internal standard peptides APASVKVFGSPTSAEV*ARVLMCLF and LVDAGKTPLLAAWAER*FVEVEA were present before but absent after tryptic digestion (Fig. 3a, c), while the product tryptic fragments of VFGSPTSAEV*AR and TPLLAAWAER* were absent before but present after tryptic digestion (Fig. 3b, d). The tryptic digestion of standard proteins (OsGSTF14 and OsGSTU6) or internal standard peptides was evaluated using the corresponding tryptic amount compared to the known amount of standard proteins or internal standard peptides. The digestion efficiency was 96.2–97.6 and 95.1–98.1% for standard proteins and internal standard peptides, respectively (Fig. 4), when they were spiked into the mobile phase, indicating that using the internal standard peptides containing natural flanking sequences around the cleavage sites improves quantitative accuracy. Identical approaches using winged peptides as the internal standards were recently applied to measure human cytokine proteins by Scott et al. [34], and bovine lactoferrin and α-lactalbumin in dairy products by Zhang et al. [35] and Lai et al. [36]. The good consistency suggests that winged peptides as the internal standards can best mimic the analytical behavior of intact OsGSTF14 and OsGSTU6 proteins.

The tryptic digestion efficiencies in rice sample matrix for standard proteins and internal standard peptides were also evaluated in the present study. It is noteworthy that the tryptic digestion efficiencies in rice sample matrix spiked with standard protein or internal standard peptide were significantly decreased compared with that in the mobile phase, indicating that the rice sample matrix has an inhibitory action on trypsin digestion efficiency. The possible reason for this is that plant tissues usually contain high concentration of phenolic compounds such as phenolic acids, which can react with the trypsin enzyme and interfere with subsequent protease digestion [37]. To avoid this, phenolic compounds are commonly removed from proteins using TCA-acetone extraction or phenol extraction protocol [38]. Although these protein extraction protocols have been successfully applied to proteomic analysis of plant tissues, some proteins are inevitably lost in multistep experiments that adversely affect target protein quantitation. Therefore, in the present study, a “minimum-step” protein extraction procedure was used. Rice roots powder was suspended in lysis buffer containing 8 M Urea, 10 mM DTT, 1 mM PMSF, 2 mM EDTA, and 20 mM Tris–HCl (pH 8.5), and vortexed and sonicated at 4 °C to destroy the structure of cell membrane and liberate the cellular proteins, then a known concentration of winged peptides as the internal standards was added into the protein lysis buffer before tryptic digestion. Compared with traditional TCA-acetone extraction or phenol extraction protocol, “minimum-step” protein extraction procedure in the present study had higher protein extraction efficiency, as reflected by their differential values in amount of extracted total proteins (Additional file 2). Moreover, the interfere effect of secondary compounds in rice sample matrix on tryptic digestion efficiency can be corrected by using winged peptides as the internal standards, because the winged peptides were combined with the target protein before tryptic digestion.

### Method validation

#### Matrix effects

**Fig. 5:**
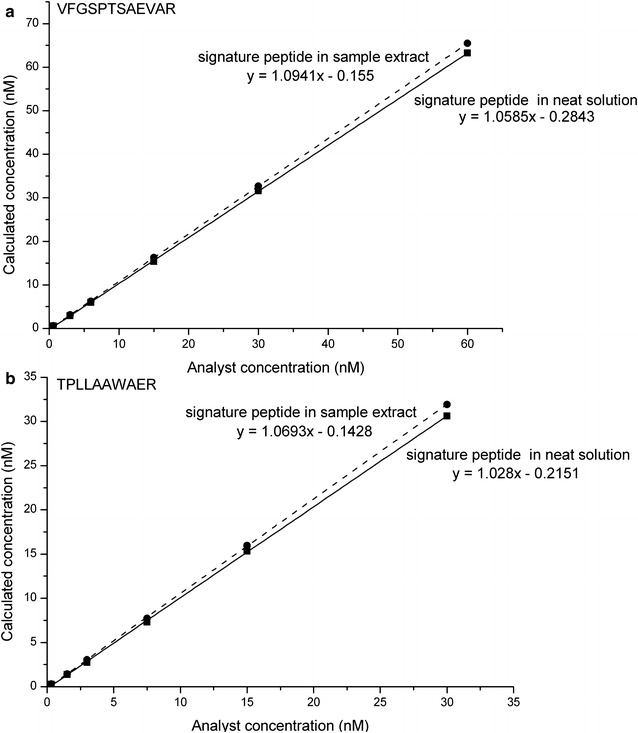
Comparison of responses of the signature peptides VFGSPTSAEVAR (**a**) and TPLLAAWAER (**b**) dissolved in neat solution to responses of the signature peptides spiked into sample extract

Matrix effects were assessed by comparing responses of the signature peptides dissolved in neat solution to responses of the signature peptides spiked into sample extract according to Matuszewski et al. [39]. The signature peptides VFGSPTSAEVAR and TPLLAAWAER from neat solution and rice sample extract showed similar linearity response in the range of 0.6–60 and 0.3–30 nM, respectively, and the response ratio of signature peptides in neat solution to that in rice sample extract were close to 1 (0.97 and 0.96) (Fig. 5). These results indicated that there were no interferences from the matrix components on the ion response of the signature peptide standards.

#### Linearity and sensitivity

Linearity was evaluated by signature peptides/isotope-labeled signature peptides area ratio (y) versus signature peptides concentration (x) in the ranges of 0.6–60 and 0.3–30 nM, respectively. The area ratio of VFGSPTSAEVAR/VFGSPTSAEV*AR exhibited good linearity in the range of 0.6–60 nM, with a linear regression equation of y = 0.094x + 0.0041 (n = 3) and correlation coefficient (r) of the standard curve greater than 0.999. Similarly, the area ratio of TPLLAAWAER/TPLLAAWAER* also exhibited good linearity in the range of 0.3–30 nM, with linear regression equation of y = 0.425x − 0.0486 (n = 3) and correlation coefficient (r) of the standard curve greater than 0.999.

Sensitivity was determined by evaluation of the limit of detection (LOD) and limit of quantitation (LOQ). The LOD and LOQ were the concentrations of the analytes at which its signal-to-noise ratios were detected as 3:1 and 10:1, respectively. They were determined by serial dilution of sample solutions under the described LC–MS/MS conditions. The LOD and LOQ were 4.5 and 14.5 µg/g for OsGSTF14, respectively, and correspondingly 2.1 and 7.0 µg/g for OsGSTU6. The sensitivity could fully meet the quantification requirements of GSTs proteins in rice samples.

#### Recovery, intra- and inter-precision

Recovery studies were performed by employing the standard addition method. Rice samples with internal standard were respectively spiked with OsGSTF14 and OsGSTU6 standard proteins at low (equal to the original level), medium and high levels. The recovery rates were calculated as the result of the measured value minus the original level divided by the spiked value. Intra- and inter-day precision (RSD) of the method were determined by recovery experiments at low, medium and high levels (n = 6) on 6 consecutive days. The results showed that the spiking recoveries rates of OsGSTF14 and OsGSTU6 standard proteins at low, medium and high level were in the ranges of 73.3–92.1 and 72.5–93.4%, respectively, with corresponding intra- and inter-day precision of 5.5–9.1 and 4.2–10.2% (Table 2), confirming that the method has a good accurate and precision.

**Table 2:** Spiked recovery test of the present LC–MS/MS method for determination of OsGSTF14 and OsGSTU6 proteins (n = 6)

Analyte	Spiked level (µg/g FW)	Detected level (µg/g FW)	Recovery rate (%)	Intra-RSD (%)	Inter-RSD (%)
OsGSTF14	15	25.5	73.3	9.7	9.1
	45	54.1	88.0	2.8	6.2
	75	83.6	92.1	1.5	5.5
OsGSTU6	10	14.2	72.5	8.6	10.2
	30	32.6	85.3	3.1	6.4
	50	53.7	93.4	1.4	4.2

### Method application

Plant GSTs play important roles in heavy metal cellular detoxification by conjugating GSH to heavy metals [7, 8]. As a detoxification mechanism, such S-glutathionylated conjugate metabolites are compartmentalized in plant vacuoles, thereby reducing heavy metal translocation and accumulation in rice grains. Previous studies demonstrated that individual rice GSTs were differentially induced by heavy metal exposure. For example, Chakrabarty et al. [15] and Dubey et al. [16] revealed the role of *OsGSTU6* in the As(V) and Cr detoxification mechanisms. Similarly, Jain et al. [17] suggested that *OsGSTF5* possibly regulates rice tolerance to As(V) stress. However, these studies mainly concentrated at the mRNA transcript level, and little information is available for changes in protein concentration of rice GSTs under heavy metal stress.

**Fig. 6:**
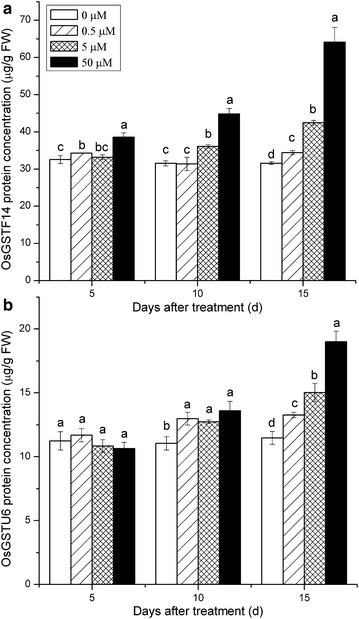
The temporal and dose responses of OsGSTF14 (**a**) and OsGSTU6 (**b**) proteins in Cd-stressed rice roots. *Letters* *a*–*d* indicate the difference among Cd treatments is significant at 0.05 level

In the present study, an optimized and advanced LC-MS/MS-based targeted proteomics assay was applied to determine the temporal and dose responses of OsGSTF14 and OsGSTU6 proteins in Cd-stressed rice roots. Cd exposure increased OsGSTF14 and OsGSTU6 protein concentrations in rice roots (Fig. 6), although the altering extent was temporal and dose-dependent. After 5 days of Cd exposure, the concentration of OsGSTF14 in roots was significantly enhanced by 0.5–50 μM Cd exposure compared with the control; in contrast, no significant changes in root OsGSTU6 concentration were detected at 0.5–50 μM Cd exposure. After Cd exposure for 10 and 15 days, both OsGSTF14 and OsGSTU6 concentrations in roots markedly increased with enhancing Cd concentrations, but the extent of increase caused by Cd exposure differed remarkably between OsGSTF14 and OsGSTU6. For example, high Cd exposure (50 μM) resulted in 1.4- (from 31.5 to 44.8 µg/g) and 2.0-fold (from 31.6 to 64.1 µg/g) increases in root OsGSTF14 concentrations compared with the control after 10 and 15 days of Cd exposure, respectively; whereas corresponding root OsGSTU6 concentrations were enhanced only 1.2- (from 11.0 to 13.6 µg/g) and 1.6-fold (from 11.5 to 19.0 µg/g). These results suggest that the OsGSTF14 and OsGSTU6 proteins possibly play important roles in the cellular detoxification mechanisms of heavy metals by conjugating GSH to Cd, which is in agreement with several previous findings that heavy metal exposure could induce marked increases in plant GSTs in rice roots [15–17]. Furthermore, OsGSTF14 protein was more susceptible to Cd exposure than OsGSTU6, as reflected by their differential changes in concentrations induced by Cd exposure, indicating OsGSTF14 is possibly mainly responsible for regulating the response of plant GSTs to Cd exposure in rice roots. In general, the assay proposed here had good accuracy, precision and high-throughput for protein quantification, which will have significant application in developing quantification methods of other proteins in Cd-stressed rice, and may provide more insight into the mechanisms of Cd translocation and accumulation in rice.

## Conclusions

In the present work, an optimized and advanced LC–MS/MS-based targeted proteomics assay for quantification of two GST members (OsGSTF14 and OsGSTU6) in Cd-stressed rice roots was developed and validated. Two synthetic tryptic signature peptides were chosen as surrogate analytes for two GSTs proteins, and winged peptides containing the isotope-labeled signature peptides were used as internal standards. The tryptic digestion efficiency of standard protein or internal standard peptides was evaluated. The assay was successfully applied to determination of the temporal and dose responses of OsGSTF14 and OsGSTU6 proteins in Cd-stressed rice roots, with good accuracy, precision and high-throughput for protein quantification. The assay proposed here will have significant application in developing quantification methods of other proteins in Cd-stressed rice, which may provide more insight into the mechanisms of Cd translocation and accumulation in rice.

## Additional files

**Additional file 1.** The SDS-PAGE and western-blot photographs of OsGSTF14 (A, B) and OsGSTU6 (C, D) standard proteins. Western blot was probed with his tag antibody, mAb.

**Additional file 2.** Comparison of protein concentrations extracted by minimum-step, TCA-acetone and phenol extraction protocols.

## Abbreviations

GSTs: glutathione transferases
GSH: tripeptide (g-Glu-Cys-Gly) glutathione
ELISA: enzyme-linked immunosorbent assay
LC–MS/MS: liquid chromatography–tandem mass spectrometry
MRM: multiple reaction monitoring
LOD: limit of detection
LOQ: limit of quantitation

## References

[1] Nordberg GF (2009). Historical perspectives on cadmium toxicology. Toxicol Appl Pharmacol.

[2] Järup L, Åkesson A (2009). Current status of cadmium as an environmental health problem. Toxicol Appl Pharmacol.

[3] Nawrot TS, Staessen JA, Roels HA, Munters E, Cuypers A, Richart T (2010). Cadmium exposure in the population: from health risks to strategies of prevention. Biometals.

[4] Satarug S, Baker JR, Urbenjapol S, Haswell-Elkins M, Reilly PE, Williams DJ (2003). A global perspective on cadmium pollution and toxicity in non-occupationally exposed population. Toxicol Lett.

[5] Tsukahara T, Ezaki T, Moriguchi J, Furuki K, Shimbo S, Matsuda-Inoguchi N (2003). Rice as the most influential source of cadmium intake among general Japanese population. Sci Total Environ.

[6] Zhang L, Gao J, Li X (2008). Chinese Total Diet Study in 2000. Cadmium intakes by different age-sex population groups. J Hyg Res.

[7] Marrs KA (1996). The functions and regulation of glutathione *S*-transferases in plants. Annu Rev Plant Biol.

[8] Cummins I, Dixon DP, Freitag-Pohl S, Skipsey M, Edwards R (2011). Multiple roles for plant glutathione transferases in xenobiotic detoxification. Drug Metab Rev.

[9] Dixon DP, Lapthorn A, Edwards R (2002). Plant glutathione transferases. Genome Biol.

[10] Soranzo N, Gorla MS, Mizzi L, De Toma G, Frova C (2004). Organisation and structural evolution of the rice glutathione *S*-transferase gene family. Mol Genet Genomics.

[11] Edwards R, Dixon DP (2005). Plant glutathione transferases. Methods Enzymol.

[12] Edwards R, Dixon DP, Cobb AH, Kirkwood RC (2000). The role of glutathione transferases in herbicide metabolism. Herbicides and their mechanisms of action.

[13] Thom R, Cummins I, Dixon DP, Edwards R, Cole DJ, Lapthorn AJ (2002). Structure of a tau class glutathione *S*-transferase from wheat active in herbicide detoxification. Biochemistry.

[14] Cho HY, Kong KH (2007). Study on the biochemical characterization of herbicide detoxification enzyme, glutathione *S*-transferase. BioFactors.

[15] Chakrabarty D, Trivedi PK, Misra P, Tiwari M, Shri M, Shukla D (2009). Comparative transcriptome analysis of arsenate and arsenite stresses in rice seedlings. Chemosphere.

[16] Dubey S, Misra P, Dwivedi S, Chatterjee S, Bag SK, Mantri S (2010). Transcriptomic and metabolomic shifts in rice roots in response to Cr(VI) stress. BMC Genomics.

[17] Jain M, Ghanashyam C, Bhattacharjee A (2010). Comprehensive expression analysis suggests overlapping and specific roles of rice glutathione *S*-transferase genes during development and stress responses. BMC Genomics.

[18] Anderson L, Seilhamer J (1997). A comparison of selected mRNA and protein abundances in human liver. Electrophoresis.

[19] Gygi SP, Rochon Y, Franza BR, Aebersold R (1999). Correlation between protein and mRNA abundance in yeast. Mol Cell Biol.

[20] Wittmann-Liebold B, Graack HR, Pohl T (2006). Two-dimensional gel electrophoresis as tool for proteomics studies in combination with protein identification by mass spectrometry. Proteomics.

[21] Rabilloud T, Chevallet M, Luche S, Lelong C (2010). Two-dimensional gel electrophoresis in proteomics: past, present and future. J Proteomics.

[22] Mahmood T, Yang PC (2012). Western blot: technique, theory, and trouble shooting. N Am J Med Sci.

[23] Gan SD, Patel KR (2013). Enzyme immunoassay and enzyme-linked immunosorbent assay. J Invest Dermatol.

[24] Linscheid MW, Ahrends R, Pieper S, Kühn A (2009). Liquid chromatography–mass spectrometry-based quantitative proteomics. Proteomics.

[25] Xie F, Liu T, Qian WJ, Petyuk VA, Smith RD (2011). Liquid chromatography–mass spectrometry-based quantitative proteomics. J Biol Chem.

[26] Wasinger VC, Zeng M, Yau Y (2013). Current status and advances in quantitative proteomic mass spectrometry. Int J Proteomics.

[27] Yang T, Xu F, Xu J, Fang D, Yu Y, Chen Y (2013). Comparison of liquid chromatography–tandem mass spectrometry-based targeted proteomics and conventional analytical methods for the determination of P-glycoprotein in human breast cancer cells. J Chromatogr B.

[28] Xu F, Yang T, Fang D, Xu Q, Chen Y (2014). An investigation of heat shock protein 27 and P-glycoprotein mediated multi-drug resistance in breast cancer using liquid chromatography–tandem mass spectrometry-based targeted proteomics. J Proteomics.

[29] Fallon JK, Smith PC, Xia CQ, Kim MS (2016). Quantification of four efflux drug transporters in liver and kidney across species using targeted quantitative proteomics by isotope dilution nanoLC–MS/MS. Pharm Res Dordr.

[30] Yoshida S, Forno DA, Cock JH, Gomez KA (1976). Laboratory manual for physiological studies of rice.

[31] Wisniewski JR, Zougman A, Nagaraj N, Mann M (2009). Universal sample preparation method for proteome analysis. Nat Methods.

[32] Annesley TM (2003). Ion suppression in mass spectrometry. Clin Chem.

[33] Gerber SA, Rush J, Stemman O, Kirschner MW, Gygi SP (2003). Absolute quantification of proteins and phosphoproteins from cell lysates by tandem MS. Proc Natl Acad Sci USA.

[34] Scott KB, Turko IV, Phinney KW (2015). Quantitative performance of internal standard platforms for absolute protein quantification using multiple reaction monitoring-mass spectrometry. Anal Chem.

[35] Zhang J, Lai S, Cai Z, Chen Q, Huang B, Ren Y (2014). Determination of bovine lactoferrin in dairy products by ultra-high performance liquid chromatography–tandem mass spectrometry based on tryptic signature peptides employing an isotope-labeled winged peptide as internal standard. Anal Chim Acta.

[36] Lai SY, Zhang JS, Zhang Y, Chen Q, Huang BF, Ren YP (2015). A combined tryptic peptide and winged peptide internal standard approach for the determination of α-lactalbumin in dairy products by ultra high performance liquid chromatography with tandem mass spectrometry. J Sep Sci.

[37] Rohn S, Rawel HM, Kroll J (2002). Inhibitory effects of plant phenols on the activity of selected enzymes. J Agric Food Chem.

[38] Isaacson T, Damasceno CM, Saravanan RS, He Y, Catalá C, Saladié M (2006). Sample extraction techniques for enhanced proteomic analysis of plant tissues. Nat Protoc.

[39] Matuszewski BK, Constanzer ML, Chavez-Eng CM (2003). Strategies for the assessment of matrix effect in quantitative bioanalytical methods based on HPLC–MS/MS. Anal Chem.

